# Evaluation of Newborn Direct Bilirubin As Screening for Cholestatic Liver Disease

**DOI:** 10.1097/PG9.0000000000000345

**Published:** 2023-08-21

**Authors:** Rikah Lerer, Lily Barash, Suhas Nafday, Debora Kogan Liberman, Nadia Ovchinsky

**Affiliations:** From the * Division of Gastroenterology, Hepatology, and Nutrition, Cohen Children’s Medical Center, Queens, NY; † Division of Gastroenterology, Hepatology, and Nutrition, Children’s Hospital at Montefiore, Bronx, NY; ‡ Division of Neonatology, Children’s Hospital at Montefiore, Bronx, NY.

**Keywords:** biliary atresia, direct hyperbilirubinemia, liver transplantation

## Abstract

**Background:**

Biliary atresia (BA) remains the most common indication for pediatric liver transplantation. Early diagnosis is essential for a favorable long-term prognosis for patients with BA. Preliminary data suggests that measurement of direct bilirubin (DB) in newborns may be an effective screening tool for neonatal cholestasis, particularly BA, allowing for early referral and diagnosis. The objective of our study was to establish a cutoff DB value to predict diagnosis of cholestatic liver disease (CLD) with high sensitivity and specificity, as well as, to evaluate whether newborns with elevated DB received appropriate follow-up in our health system.

**Methods:**

Baseline data were collected on infants born between 2016 and 2019 who had serum total bilirubin and DB drawn in the nursery, and who continued to follow in our health system. Sensitivity, specificity, and positive and negative predictive values were examined using cutoff values of 0.5, 0.6, and 0.7 mg/dL for identifying infants at risk for CLD. Patients’ charts were reviewed to note whether they had follow-up levels drawn by their pediatrician or by the hepatology team within 2 months of age and whether they were diagnosed with CLD.

**Results:**

Serum total bilirubin and DB levels were drawn from 11 965 infants during their hospitalizations. Three infants from this cohort were diagnosed with CLD: 2 with BA and 1 with Alagille syndrome. DB cutoff values of 0.5, 0.6, and 0.7 mg/dL had sensitivity of 100% and specificity of 96.83% (95% confidence interval [CI], 96.69%-97.53%), 99.08% (95% CI, 98.81%-99.30%), and 99.63% (95% CI, 99.4%-99.7%), respectively. Given that a DB of 0.6 mg/dL had a sensitivity of 100% and specificity of 99%, this value was chosen as the cutoff value to monitor for DB follow-up and diagnosis of CLD. Out of 60 infants who met criteria for DB ≥0.6 mg/dL, only 15 (25%) had a repeat level drawn after nursery discharge; 3 (5%) were eventually diagnosed with CLD.

**Conclusions:**

A DB cutoff value of 0.6 mg/dL yielded high sensitivity and specificity for identifying patients with CLD. All 3 patients diagnosed with CLD had elevated DB at hospital discharge. The data revealed that the majority (75%) of eligible newborns did not receive follow-up for their elevated DB in the outpatient setting.

**What Is Known**

- Early diagnosis is essential for a favorable long-term prognosis for patients with biliary atresia.
- Diagnosis is often delayed due to lack of a universal screening program in the United States.
- Newborn serum bilirubin levels may be an effective screening tool.

**What Is New**

- Direct bilirubin in newborns is a serum biomarker that is sensitive and specific for cholestatic liver disease.
- Direct bilirubin screening in newborns may help identify biliary atresia and other cholestatic liver diseases earlier.

## INTRODUCTION

Cholestatic liver diseases (CLDs) are rare disorders that affect the anatomy or function of the bile canaliculi and duct pathway, characterized by chronic cholestasis and jaundice (1). Biliary atresia (BA) remains the most common CLD in children and the leading indication for pediatric liver transplantation. The initial treatment for BA involves performing a Kasai portoenterostomy (KP) (2). If not treated early, it progresses to severe liver injury; if untreated, it causes death by 2 years of age. Ultimately, the majority of patients will need a liver transplant (3). It is increasingly accepted that KP at ≤30 days of life significantly improves the native liver survival rate (3–5).

The challenge to diagnosing BA early is that there are neither specific early biological markers for screening nor a widely accepted screening approach (2,6). The first clinical sign may be jaundice, which can be a symptom of many possible diagnoses. When an infant presents with jaundice, the work-up can take weeks to confirm a diagnosis of BA and by then, it might be too late to effectively perform a KP. Early diagnosis also improves outcomes in other conditions associated with neonatal cholestasis, mainly by preventing the development of malnutrition and fat-soluble vitamin deficiencies (7–10).

There is growing evidence that newborn serum bilirubin levels may be effective as a screening tool, given that all infants with BA exhibit direct hyperbilirubinemia at birth (2,4–6,10–16). Two retrospective studies performed at large medical centers showed that infants born between 2007–2010 and 2009–2011 with BA had elevated conjugated or direct bilirubin (DB) at birth (2,12). A subsequent prospective study at the same center showed that a 2-stage screening with direct or conjugated bilirubin measurements could significantly reduce the age at which infants underwent KP from 56 days before screening implementation to 36 days after screening implementation (17).

In a 2015 technical report, the American Academy of Pediatrics advocated to study the implementation of newborn screening for BA in the United States. Currently, the American Academy of Pediatrics recommends checking total bilirubin (TB) and DB if a newborn is jaundiced after 2 weeks of age, except in the case of breastfeeding infants; then the screening can wait until 3 weeks of age (18). This could cause a delay in screening, especially in places where there is no standard 1-month pediatric visit.

As the role of newborn bilirubin screening is increasingly recognized, the goals of our study were to determine whether neonates with elevated DB received appropriate follow-up within our health system as well as to establish a cutoff DB value to predict diagnosis of CLD with high sensitivity and specificity.

## METHODS

A multidisciplinary team involving representatives from Pediatrics, Neonatology, and Hepatology was established. The project was approved by the institutional review board at Albert Einstein College of Medicine. Data collection involved a retrospective chart review of infants born at Wakefield and Weiler Hospitals, the 2 major delivery sites within Montefiore Medical Center (MMC), a tertiary care center with multiple locations in the Bronx and Westchester in New York.

### Study Population

Baseline data were collected on infants born between 2016 and 2019, who had measurements of DB and continued to follow within the MMC health system. Blood work in the nursery was mainly done to assess physiologic jaundice risk. In 2016, Epic electronic medical record was first introduced at all MMC campuses, which united the nurseries and allowed this data to be reviewed. All infants ≥34 weeks gestation who either were admitted to the newborn nursery or had a short neonatal intensive care unit (NICU) stay (defined as <96 hours) were included. Premature infants and those with a prolonged NICU stay were excluded to minimize confounding factors that could influence bilirubin levels. Infants with prolonged NICU stays often experienced ischemic events, had infections, received total parenteral nutrition, or had limited enteral nutrition. These infants had long-term follow-up with appropriate evaluation of cholestasis while in the NICU.

### Screening

Bilirubin assays are analyzed using Abbott Architect c8000. Both TB and DB are automatically generated as standard laboratory practice. The upper limit of normal is determined by previous reference range studies. The upper limit of normal for DB in our health system is 0.4 mg/dL.

We examined the sensitivity, specificity, positive predictive value, and negative predictive value of values just above the laboratory reference range upper limit of normal using 0.5, 0.6, and 0.7 mg/dL as cutoff values for identifying infants at risk for CLD. Both initial and discharge bilirubin were investigated. Although the sensitivity between using the first versus the discharge bilirubin was comparable, the specificity of using discharge bilirubin is greater (Tables 1–3).

We chose to use the discharge DB as our cutoff due to better specificity to limit outpatient follow-up to children who had persistently elevated DB or rising DB. Children with BA all had rising DB on repeat measurements. The average timing of a discharge DB was at 2.57 days of life.

Additionally, we examined the charts of patients with BA who were followed at our institution to determine their lowest DB value that was detected in the nursery and if it had been measured.

### Data Collection

An excel spreadsheet was used to manually record infants who were born during that time frame, their initial DB, and their DB prior to discharge from the nursery. Newborn and subsequent general pediatrician visits until 3 months of age were reviewed to record any follow-up DB values that were obtained or any referrals that were made to a hepatologist. If found, the diagnosis of CLD and the timeline of diagnosis were recorded. The charts of patients with BA who received a KP at MMC during the study period were reviewed to ensure no children were missed during the screening evaluation.

## RESULTS

Between 2016 and 2019, there were 22 465 total births within MMC. Of these, 11 965 newborns had TB and DB levels drawn during their hospitalization.

Three infants from this cohort were diagnosed with CLD: 2 with BA and 1 with Alagille syndrome.

### Screening Data

Prior to hospital discharge, 181 infants had a DB ≥0.5 mg/dL, 60 infants had a DB ≥0.6 mg/dL, and 26 infants had a DB ≥0.7 mg/dL (Table 2). The sensitivity for all 3 values was 100% (95% confidence interval [CI], 29.24%-100%). The specificity was 96.83% (95% CI, 96.69%-97.53%), 99.08% (95% CI, 98.81%-99.30%), and 99.63% (95% CI, 99.4%-99.7%) for 0.5, 0.6, and 0.7 mg/dL, respectively (Table 1).

**TABLE 1.** Newborn direct bilirubinemia screening for cholestatic liver disease

	% (95% CI)		
	≥0.5 mg/dL	≥0.6 mg/dL	≥0.7 mg/dL
Sensitivity	100% (29.24%-100.0%)	100% (29.24%-100%)	100% (29.24%-100%)
Specificity	96.83% (96.69%-97.53%)	99.08% (98.81%-99.30%)	99.63% (99.4%-99.77%)
PPV	1.7% (0.4%-5.1%)	5% (1.3%-14.8%)	11.5% (3.0%-31.3%)
NPV	100% (99.92%-100%)	100% (99.92%-100.0%)	100% (99.92%-100.0%)

CI = confidence interval; NPV = negative predictive value; PPV = positive predictive value.

**TABLE 2.** Cutoff direct bilirubin values for cholestatic liver disease

Discharge DB	CLD	Negative
DB ≥0.5 mg/dL	3	178
DB <0.5 mg/dL	0	6033
DB ≥0.6 mg/dL	3	57
DB <0.6 mg/dL	0	6154
DB ≥0.7 mg/dL	3	23
DB <0.7 mg/dL	0	6188

CLD = cholestatic liver disease; DB = direct bilirubin.

We found that higher DB levels at our center may yield a higher positive predictive value and lower number of patients would screen positive. However, these values are specific to our center and may not be representative of other centers. Previous studies at a larger health system used a much lower level of 0.4 mg/dL for their screening process (17). Therefore, to capture all children with CLD, 0.6 mg/dL was chosen as the cutoff value to monitor for follow-up and diagnosis of CLD. This value provided sensitivity of 100% and specificity of 99%. In addition, chart review of 23 BA patients who had DB values available at our liver center revealed that no infant had a DB value <0.6 mg/dL at birth.

**FIGURE 1.**
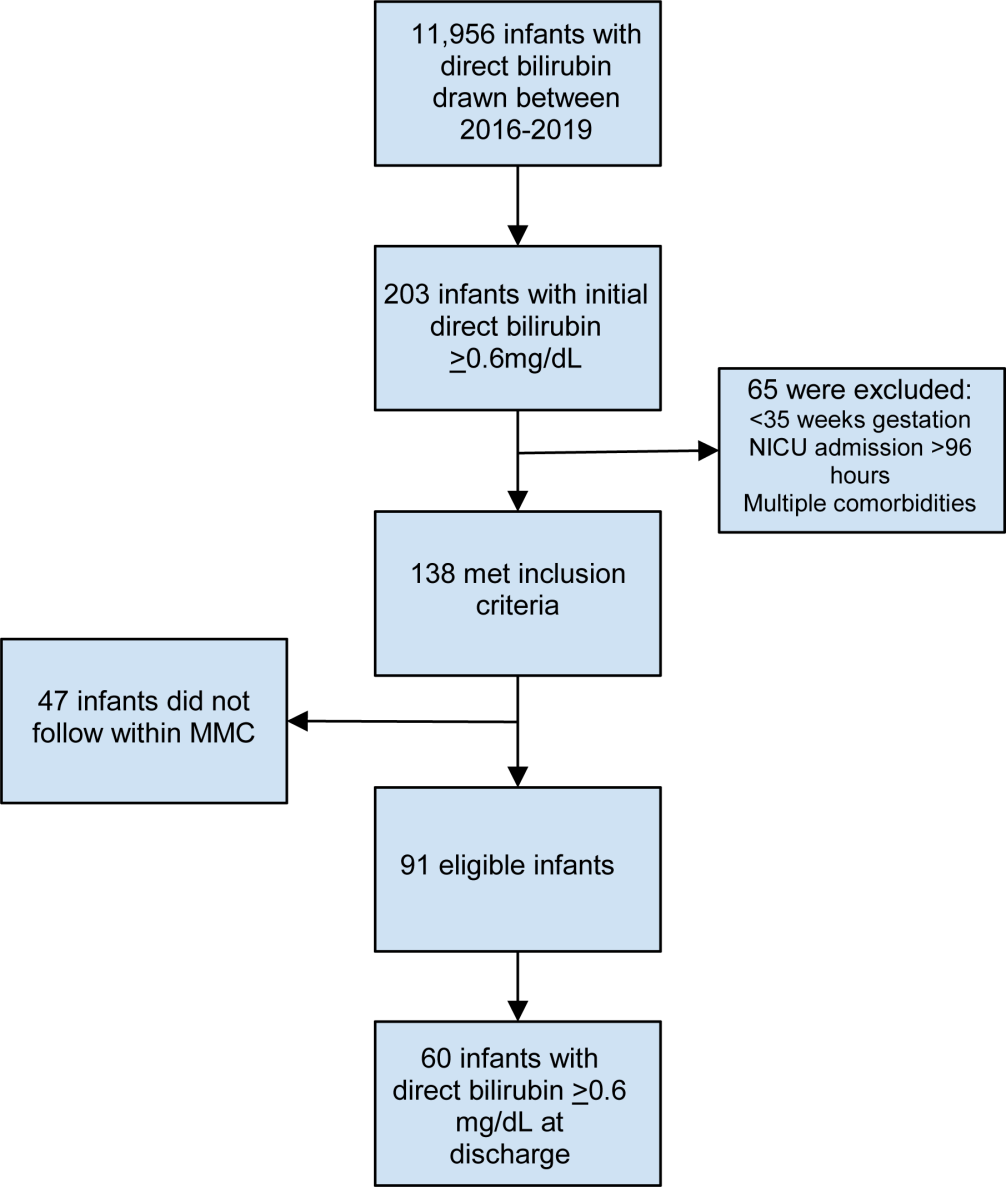
Study population. MMC = Montefiore Medical Center; NICU = neonatal intensive care unit.

**FIGURE 2.**
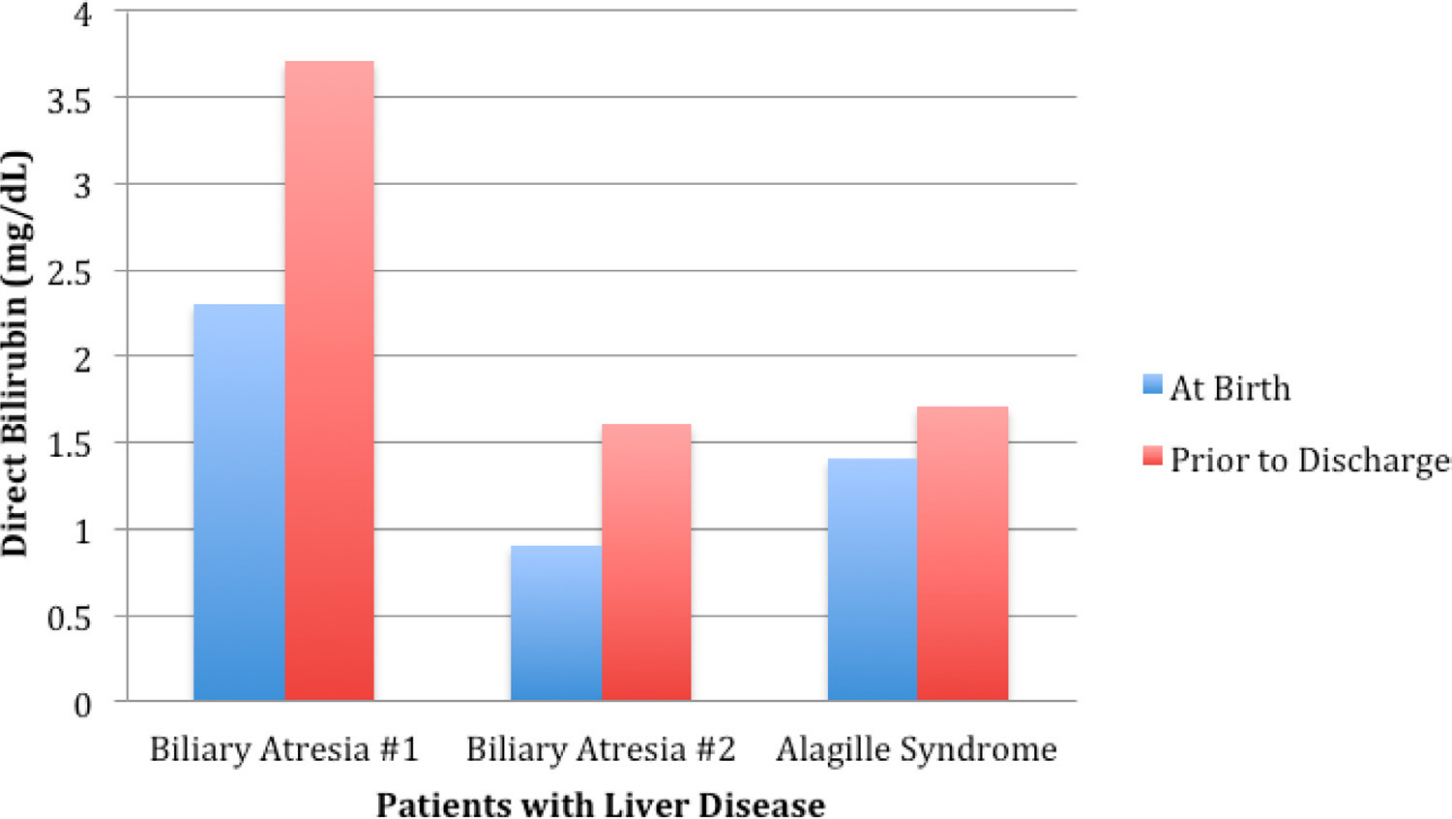
Direct bilirubin levels at birth and pre-discharge in newborns eventually diagnosed with liver disease at MMC. MMC = Montefiore Medical Center.

We further examined our cohort using the DB cutoff value of 0.6 mg/dL. Sixty infants met criteria and had a DB ≥0.6 mg/dL at nursery discharge (Fig. 1). Of 60 infants with DB ≥0.6 mg/dL, only 15 (25%) had a repeat level drawn after nursery discharge by their primary pediatrician or by a hepatologist. Three (5%) infants were diagnosed with CLD. The diagnosis of BA in the 2 infants was made at the age of 23 and 25 days. All infants with CLD showed rising DB values on repeat testing prior to discharge (Fig. 2). Charts of the infants without DB follow-up were also reviewed up until 3 months of age; those infants did not present later with any liver pathology. Discharge DB values for cholestatic infants with DB ≥0.6 mg/dL without follow-up can be found in Supplementary Table 2 (http://links.lww.com/PG9/A133). TB and DB values prior to discharge for all infants with DB >0.6 mg/dL with DB:TB ratio can be found in Supplementary Table 3 (http://links.lww.com/PG9/A134).

Screening using the DB cutoff of 0.6 mg/dL identified the 3 known infants with CLD in the study population. All infants with true positive screening had elevated newborn DB values. Fifty-seven infants tested positive in screening but did not have CLD. Of the infants who had DB drawn after nursery discharge, 12 were not diagnosed with a CLD. On subsequent testing and follow-up, cholestasis resolved in this group of infants. They had either normalized or down trending DB within 2 weeks of life (Supplementary Table 1, http://links.lww.com/PG9/A132). This is in contrast to the infants with BA, who had a rise in DB within the first 2 weeks of life. One of these infants was identified to have a heterozygous mutation in alpha-1 antitrypsin gene.

## DISCUSSION

Preliminary data suggests that using DB values in newborns may be an effective screening tool for BA and other CLDs. Retrospective review at MMC confirmed high sensitivity (100%) and specificity (99%) when 0.6 mg/dL is used as a DB cutoff for screening newborns for CLD. All 3 patients diagnosed with a CLD had an elevated DB in the newborn period with significant rise within the first 2 weeks of life, whereas infants without CLD showed down trending values within the same time period. This suggests a predictive trend in infants eventually diagnosed with CLD.

Our retrospective data also highlights that the majority of eligible infants with elevated DB levels did not receive follow-up with a repeat DB measurement. This data led to a quality improvement initiative within our health system to establish an algorithm for the follow-up of newborns with direct hyperbilirubinemia with the ultimate goal of earlier diagnosis of CLD.

Our data is limited by the follow-up rate within MMC after nursery discharge. About 50% of newborns received care outside of MMC and therefore were excluded, as we could not access their medical records. A quality improvement project has been implemented to address this and to enhance referrals from these outside practices to our liver center.

It has become more widely accepted that performing a KP at an earlier age, especially within 30 days of life, significantly improves native liver survival rates (3–5). Other cholestatic disorders such as alpha-1 antitrypsin deficiency, Alagille syndrome, and progressive familial intrahepatic cholestasis can also benefit from earlier diagnosis and therapeutic interventions in the prevention of issues such as malabsorption and fat-soluble vitamin deficiencies (6–9). Currently, infants with CLD may not be identified until later physical findings of significant jaundice, acholic stools, or hepatosplenomegaly are present, which can delay the onset of work-up and diagnosis. Although BA meets disease-specific criteria warranting newborn screening, there is currently no widely accepted screening approach for BA in the United States (18). A promising large prospective study done in the United States showed that a 2-stage screening with direct or conjugated bilirubin measurements could significantly reduce the age at which infants underwent KP from 56 days before screening implementation to 36 days after screening implementation (17).

Studies evaluating cost-effectiveness of DB screening are limited. A recent study in Japan evaluated event-free life-years defined as liver transplant free survival, costs, and incremental cost-effectiveness (ICER) over a 25-year period. DB compared with no screening resulted in ICER of 400 229 per event-free life-years gained. They also found that DB testing could be cost-effective in Japan depending on willingness-to-pay thresholds, which vary widely across countries. DB screening did result in 18 fewer liver transplants than no screening (19). A study in Canada similarly found an ICER for DB screening to be 473 840 per life-year gained (20). Further studies are needed to first determine optimal screening values to test for BA. Then, the effectiveness and cost-effectiveness of implementing this program can be properly evaluated.

In our system, there was no additional cost to retrospectively evaluate DB trends. Fractionated bilirubin is automatically generated at many centers and can be used to retrospectively evaluate proper DB screening measures without cost.

Interventions are currently being implemented at MMC to ensure timely follow-up of direct hyperbilirubinemia. This quality improvement project will aim to better assess follow-up of DB in the community. Interventions include the education of nursery providers and outpatient pediatricians, the establishment of a referral system to Hepatology for all identified newborns with a DB ≥0.6 mg/dL at nursery discharge, and the creation of a smart phrase in the electronic medical record to be included in the newborn discharge summary. Since infants with CLD showed rising DB values on repeat blood work, it was decided to prospectively screen based on their discharge value from the nursery/NICU. Data is being collected on the rates of a follow-up DB after nursery discharge and the timing of diagnosis of BA and other CLDs. Prospectively we are now investigating DB ≥0.7 mg/dL as a cutoff value.

## CONCLUSIONS

In conclusion, neonatal DB is a serum biomarker that is both sensitive and specific for CLD. In our retrospective chart review, DB was elevated in newborns who were later diagnosed with liver disease. The majority of newborns with elevated DB do not receive follow-up for repeat DB measurement. Further studies are needed to standardize the follow-up for these infants and to improve cost-effectiveness of testing and implementation of serum bilirubin as a national screening for CLD. The DB cutoff of 0.6 mg/dL is site specific and based on the upper limit of normal at our institution. Each center should derive upper limits for DB testing which is laboratory specific. Many institutions are automatically fractioning bilirubin which can allow multicenter testing of validity and reproducibility of appropriate DB screening values.

**TABLE 3.** Cutoff direct bilirubin values for cholestatic liver disease using initial bilirubin

Initial DB drawn:	Liver disease +	No disease	Sensitivity	1	PPV	0.011
Bilirubin ≥0.5	3	251				
<0.5	0	5960	Specificity	0.95	NPV	1
0.6 Data	Liver disease +	No disease	Sensitivity	1	PPV	0.032
Bilirubin ≥0.6	3	89				
<0.6	0	6122	Specificity	0.98	NPV	1
0.7 Data	Liver disease +	No disease	Sensitivity	1	PPV	0.06
Bilirubin ≥0.7	3	41				
<0.7	0	6170	Specificity	0.99	NPV	1

DB = direct bilirubin; NPV = negative predictive value; PPV = positive predictive value.

## Supplementary Material


