# Supplementary material for: Evaluation of Newborn Direct Bilirubin As Screening for Cholestatic Liver Disease
Source: JPGN Rep. 2023 Aug 21;4(4):e345. doi: 10.1097/PG9.0000000000000345 (PMC10684158; doi:10.1097/PG9.0000000000000345)
Supplement: Supplementary file 1 [file pg9-4-e345-s001.pdf]

Supplementary table 1: Trend of direct bilirubin for cholestatic infants with DB drawn after nursery discharge without chronic liver disease.

**Initial Direct Bilirubin (mg/dL)**	**Discharge Direct Bilirubin (mg/dL)**	**Peak follow-up direct bilirubin (mg/dL)**	**Follow-up Provider**
0.8	0.8	0.3	Pediatrician
0.9	0.8	1	Pediatrician
0.6	0.6	0.5	Pediatrician
0.8	0.8	0.5	Pediatrician
1.2	1.2	0.3	Gastroenterologist
1.2	0.7	0.3	Pediatrician
0.8	0.8	2.1*	Pediatrician
1.9	1.5	0.1	Hepatologist
1	1.9	2.2**	Pediatrician
1	1	0.6	Pediatrician
1.5	1.2	0.4	Hepatologist
1.4	1.2	2.3***	Pediatrician

*- At DOL 14, DB down trending to 0.9mg/dL
**- At DOL 15, DB down trending to 1.8mg/dL
***- At DOL 13 DB down trending to 1.3 mg/dL
