# Supplementary material for: Evaluation of Newborn Direct Bilirubin As Screening for Cholestatic Liver Disease
Source: JPGN Rep. 2023 Aug 21;4(4):e345. doi: 10.1097/PG9.0000000000000345 (PMC10684158; doi:10.1097/PG9.0000000000000345)
Supplement: Supplementary file 2 [file pg9-4-e345-s002.pdf]

Supplementary Table 2: Direct bilirubin values for cholestatic infants without repeat direct bilirubin drawn after discharge

Initial DB (mg/dL)	DB prior to discharge (mg/dL)
0.6	0.6
0.7	0.7
1	1
0.6	0.7
0.6	0.6
1	1
0.6	0.6
0.6	0.6
0.6	0.6
0.6	0.7
1	1
0.7	0.6
0.6	0.6
1	0.8
0.8	0.6
0.7	1
0.8	0.7
0.9	0.9
0.6	1.4
0.9	0.9
0.6	0.6
0.6	0.6
0.6	0.6

0.6	0.6
0.6	0.6
0.7	0.7
0.6	0.6
0.6	0.6
0.7	0.7
0.6	0.6
1.3	0.6
0.6	0.6
0.6	0.6
0.7	0.8
0.6	0.7
0.6	0.6
0.6	0.6
0.6	0.6
0.6	0.6
0.6	0.6
0.7	0.6
0.6	0.6
0.6	0.6
0.6	0.6
0.6	0.6
