# Supplementary material for: Evaluation of Newborn Direct Bilirubin As Screening for Cholestatic Liver Disease
Source: JPGN Rep. 2023 Aug 21;4(4):e345. doi: 10.1097/PG9.0000000000000345 (PMC10684158; doi:10.1097/PG9.0000000000000345)
Supplement: Supplementary file 3 [file pg9-4-e345-s003.pdf]

Supplementary Table 3: Discharge Total Bilirubin and Direct Bilirubin with ratio

**Discharge TB (mg/dL)**	**Discharge DB (mg/dL)**	**Ratio DB:TB**	**Received follow up?**
9.2	0.6	0.065	no
9.3	0.7	0.075	no
10.4	1.7	0.163	yes**
9.4	1	0.106	no
2.6	0.7	0.269	no
9.2	0.6	0.065	no
5.1	1	0.196	no
2	0.6	0.3	no
8	0.6	0.075	no
3.2	0.8	0.25	yes
6.7	0.6	0.089	no
2.4	0.7	0.291	no
8.7	0.8	0.091	yes
11.3	1	0.088	no
1.5	0.6	0.4	no
9.1	0.6	0.065	no
4	0.8	0.2	no
12.4	0.6	0.048	yes
4.7	0.8	0.170	yes
7.2	0.6	0.083	no
2.9	1.2	0.413	yes
10.4	1	0.096	no
7.2	0.7	0.097	no
3.1	0.9	0.290	no
10.5	1.8	0.171	yes*

1.9	0.7	0.368	yes
10.5	1.4	0.133	no
2.6	0.9	0.346	no
4.9	0.6	0.122	no
8.6	0.6	0.069	no
7.1	0.8	0.112	yes
6.6	0.6	0.090	no
5.6	1.5	0.267	yes
8.1	0.6	0.074	no
7.7	0.6	0.077	no
10.7	1.9	0.177	yes
8.9	0.7	0.078	no
9.2	0.6	0.065	no
5.2	0.6	0.115	no
4.8	0.7	0.145	no
10.4	0.6	0.057	no
1.3	0.6	0.461	no
8.6	0.6	0.069	no
7.7	0.6	0.077	no
8.5	0.8	0.094	no
10.4	0.7	0.067	no
9.8	0.6	0.061	no
1.6	0.6	0.375	no
4.2	1	0.238	yes
9.4	0.6	0.063	no
8.6	0.6	0.069	no
14.8	3.7	0.25	yes*
5.6	0.6	0.107	no

2.6	1.2	0.461	yes
6.3	0.6	0.095	no
9.5	0.6	0.063	no
2.5	0.6	0.24	no
9.1	0.6	0.065	no
10.1	1.2	0.118	yes
10.4	0.6	0.057	no

*- Biliary atresia

**- Alagille syndrome
